# Mitochondrial H_2_O_2_ Is a Central Mediator of Diclofenac-Induced Hepatocellular Injury

**DOI:** 10.3390/antiox13010017

**Published:** 2023-12-21

**Authors:** Sin Ri Kim, Ji Won Park, You-Jin Choi, Seong Keun Sonn, Goo Taeg Oh, Byung-Hoon Lee, Tong-Shin Chang

**Affiliations:** 1 Graduate School of Pharmaceutical Sciences, Ewha Womans University, Seoul 03760, Republic of Korea; 2 College of Pharmacy and Research Institute of Pharmaceutical Sciences, Seoul National University, Seoul 08826, Republic of Korea; 3 Heart-Immune-Brain Network Research Center, Department of Life Science, Ewha Womans University, Seoul 03760, Republic of Korea

**Keywords:** diclofenac, hepatotoxicity, peroxiredoxin III, reactive oxygen species, mitochondrial H_2_O_2_, mitochondrial dysfunction, apoptosis, nonsteroidal anti-inflammatory drug

## Abstract

Nonsteroidal anti-inflammatory drug (NSAID) use is associated with adverse consequences, including hepatic injury. The detrimental hepatotoxicity of diclofenac, a widely used NSAID, is primarily connected to oxidative damage in mitochondria, which are the primary source of reactive oxygen species (ROS). The primary ROS responsible for inducing diclofenac-related hepatocellular toxicity and the principal antioxidant that mitigates these ROS remain unknown. Peroxiredoxin III (PrxIII) is the most abundant and potent H_2_O_2_-eliminating enzyme in the mitochondria of mammalian cells. Here, we investigated the role of mitochondrial H_2_O_2_ and the protective function of PrxIII in diclofenac-induced mitochondrial dysfunction and apoptosis in hepatocytes. Mitochondrial H_2_O_2_ levels were differentiated from other types of ROS using a fluorescent H_2_O_2_ indicator. Upon diclofenac treatment, PrxIII-knockdown HepG2 human hepatoma cells showed higher levels of mitochondrial H_2_O_2_ than PrxIII-expressing controls. PrxIII-depleted cells exhibited higher mitochondrial dysfunction as measured by a lower oxygen consumption rate, loss of mitochondrial membrane potential, cardiolipin oxidation, and caspase activation, and were more sensitive to apoptosis. Ectopic expression of mitochondrially targeted catalase in PrxIII-knockdown HepG2 cells or in primary hepatocytes derived from PrxIII-knockout mice suppressed the diclofenac-induced accumulation of mitochondrial H_2_O_2_ and decreased apoptosis. Thus, we demonstrated that mitochondrial H_2_O_2_ is a key mediator of diclofenac-induced hepatocellular damage driven by mitochondrial dysfunction and apoptosis. We showed that PrxIII loss results in the critical accumulation of mitochondrial H_2_O_2_ and increases the harmful effects of diclofenac. PrxIII or other antioxidants targeting mitochondrial H_2_O_2_ could be explored as potential therapeutic agents to protect against the hepatotoxicity associated with NSAID use.

## 1. Introduction

Nonsteroidal anti-inflammatory drugs (NSAIDs) are among the most widely used analgesic and anti-inflammatory medications around the globe. However, their use is accompanied by a range of adverse consequences, such as gastrointestinal, cardiovascular, renal, and hepatic issues. Because of their severe cardiovascular and hepatic toxicity, certain NSAIDs have been removed from the market. According to the Drug-Induced Liver Injury Network Database, diclofenac is the NSAID most often associated with the onset of hepatocellular injury [1].

It has been demonstrated that reactive oxygen species (ROS) are essential for hepatocyte apoptosis and liver damage caused by diclofenac [2,3,4]. Mitochondria are the primary source of ROS in the hepatocytes following diclofenac exposure, and diclofenac’s harmful hepatotoxicity is primarily related to the effects of ROS on mitochondria [2,3,5]. Both diclofenac itself and its reactive metabolites affect mitochondrial activity and produce ROS, which can cause apoptosis in human and rat hepatocytes [3,5,6]. Additionally, diclofenac and metabolites impede ATP production and oxidative phosphorylation in rat liver mitochondria [5]. Notably, diclofenac inhibits the electron transport chain (ETC) complexes I and III, which could thereby lead to electron leakage from the respiratory chain, slowing mitochondrial respiration [7,8]. Hepatocytes exposed to diclofenac may experience mitochondrial malfunction and increased ROS generation. ROS can induce apoptotic signaling pathways, release of pro-apoptotic proteins, and apoptosis [4,7].

Mitochondrial ROS arise when O_2_ is reduced by one electron to form superoxide anion (O_2_^•−^). Mitochondrial ETC complexes I and III are one-electron leakage sites in diclofenac-treated cells [7,8]. Mitochondrial O_2_^•−^ can be dismutated to H_2_O_2_ either spontaneously or enzymatically by superoxide dismutase 2 (SOD2) in the mitochondrial matrix or SOD1 in the intermembrane region [9,10,11]. Uncharged H_2_O_2_ made by mitochondrial SODs can move across the mitochondrial membrane into the cytosol, increasing cytosolic H_2_O_2_. In contrast, negatively charged O_2_^•−^ is not readily able to pass through the membrane. Through the production of H_2_O_2_, a mild oxidant, the SOD reaction alleviates some of the oxidative stress caused by O_2_^•−^. Nevertheless, the Fenton reaction readily transforms H_2_O_2_ into the much more dangerous hydroxyl radical (^•^OH). Thus, among various ROS, mitochondrial H_2_O_2_ is likely to play a key role in the hepatocyte damage caused by diclofenac. To shield hepatocytes against diclofenac-induced harm, including mitochondrial malfunction and death, antioxidant enzymes that remove mitochondrial H_2_O_2_ are necessary. Although mitochondria contain H_2_O_2_-removing enzymes such as glutathione peroxidase 1 and 4, and peroxiredoxin (Prx) V, approximately 90% of mitochondrial H_2_O_2_ appears to be eliminated by PrxIII in most cell types [12].

The primary ROS responsible for diclofenac-induced hepatocellular toxicity and the principal antioxidant that mitigates this ROS are not yet known. In this study, we aimed to elucidate the major role of mitochondrial H_2_O_2_ and a protective function of PrxIII in the context of diclofenac-induced mitochondrial dysfunction and apoptosis in hepatocytes.

## 2. Materials and Methods

### 2.1. Animals

Wild-type (PrxIII^+/+^) and PrxIII-deficient (PrxIII^−/−^) mice were maintained on a C57BL/6 background [13,14]. All experiments were performed on age-matched male mice between 6 and 8 weeks of age. All animal care and experimental techniques followed Ewha Womans University’s Institutional Animal Care and Use Committee regulations.

### 2.2. Reagents and Antibodies

Reagents utilized in this study include diclofenac sodium (D6899) and puromycin (p8833) from Sigma Aldrich (St. Louis, MO, USA); the annexin V-fluorescein isothiocyanate (FITC) apoptosis kit (556547) was purchased from BD biosciences (San Jose, CA, USA); acetyl-Leu-Glu-His-Asp-7-amino-4-trifluoromethylcoumarin (Ac-LEHD-AFC) and acetyl-Asp-GluVal-Asp-7-amino-4-methylcoumarin (Ac-DEVD-AMC) were from BIOMOL (Hamburg Germany); 5-(and-6)-chloromethyl-2′,7′-dichlorodihydrofluorescein diacetate (CM-H_2_DCFDA), 10-N-nonyl-acridine orange (NAO) (A1372), and tetramethylrhodamine ethyl ester (TMRE) (T669) were from Molecular Probes ( Eugene, OR, USA); mitochondria peroxy-yellow-1 (MitoPY1) (4428) was from Tocris Biosciences; FuGene6 (E2311) and pSUPER-puro vector were from Promega (Madison, WI, USA) and OligoEngine (Seattle, WA, USA) respectively. The following antibodies were used: anti-PrxIII (LF-MA0329) and anti-β-actin (ab8226) were purchased from Abcam (Cambridge, UK); anti-catalase (LF-PA0060) was from Abfrontier (Seoul, Republic of Korea). Adenovirus expressing human catalase with a mitochondrial leader sequence (mito-Catalase) was used as described previously [14,15].

### 2.3. Cell Culture and Infection

HepG2 human hepatoma cells (ATCC, Manassas, VA, USA) were maintained in Dulbecco’s modified Eagle’s medium (DMEM; Hyclone, Logan, UT, USA, SH30021.01) supplemented with 10% FBS (Gibco, 16000044) and 1% antibiotics–antimycotics (Gibco, 15250062) at 37 °C in a humidified atmosphere containing 5% CO_2_. For infection of mito-Catalase adenovirus, HepG2 cells were seeded in 12-well plates and incubated with the adenovirus the following day for 24 h. Mouse primary hepatocyte isolation was performed as described previously [4].

### 2.4. Establishment of HepG2 Cells Expressing Small Hairpin RNA Targeting PrxIII

The small hairpin interfering RNA oligonucleotide sequences targeting human PrxIII [15] used to construct a pSUPER siPrxIII were purchased from Genotec (Daejeon, Korea), annealed, and cloned into the pSuperior-puro (pSUPER) vector. HepG2 cells were transfected with pSUPER_siPrxIII vectors using FuGene6 reagent (Promega, Madison, WI, USA). Single clones were grown and described after selection with 1.5 μg/mL puromycin.

### 2.5. Western Blotting

Cell lysates were prepared as described previously [16]. Briefly, cells were lysed in 20 mM HEPES buffer (pH 7.0) containing 150 mM NaCl, 1% Triton X-100, 2 mM EGTA, 1 mM EDTA, 20 mM β-glycerophosphate, 10% glycerol, 1 mM 4-(2-aminoethyl) benzenesulfonyl fluoride hydrochloride (AEBSF), aprotinin (10 μg/mL), and leupeptin (10 μg/mL). Cell debris was removed by centrifugation at 12,500× *g* for 10 min at 4 °C. Equal volume of cell lysates with adjusted protein concentration were subjected to Western blotting analysis using specific antibodies, as indicated. Band intensity was analyzed using ImageJ (NIH, Bethesda, MD, USA).

### 2.6. Determination of Mitochondrial H_2_O_2_

Cells in glass-bottomed 35 mm culture dishes reached 80% confluence (MatTeK, Ashland, OH, USA). Cells were stimulated, washed twice with phenol red–free culture media, and incubated with each indicator in 1 percent fetal bovine serum for 20 min at 37 °C. After replacing media with phenol red–free growth media containing 1% fetal bovine serum, the LSM 880 AiryScan (Carl Zeiss, Göttingen, Germany) captured fluorescence pictures on a temperature-controlled stage. To detect mitochondrial H_2_O_2_, cells were incubated with MitoPY1 (10 μM). The excitation/emission wavelength for MitoPY1 was 488/525 nm. Fluorescence intensity was measured and visualized using NIS-Elements software 3.1 (Nikon, Tokyo, Japan).

### 2.7. Flow Cytometry Analyses

To detect cellular ROS, detached cells were loaded with CM-H_2_DCFDA (10 μM) for 10 min at 37 °C. Mitochondrial damage was measured in cells stained with either TMRE (50 nM) or NAO (50 nM) at 37 °C for 20 min. To analyze cell death, cells were resuspended in annexin binding buffer and labeled with annexin-V-FITC and propidium iodide (PI) at 25 °C for 15 min, according to the manufacturer’s instructions (Annexin-V-FITC and PI kits; BD Biosciences, San Jose, CA, USA). Cells were analyzed using a FACSCalibur flow cytometer (BD Biosciences, San Jose, CA, USA) with excitation wavelength 488 nm and observation wavelength 530 nm for green fluorescence and 585 nm for red fluorescence. Relative change in fluorescence was analyzed with FlowJo software 10.9 (FlowJo LLC, Ashland, OR, USA).

### 2.8. Oxygen Consumption Rate (OCR) Measurement

Cells were cultured in Seahorse XFp plates at a density of 4 × 10^4^ cells/well in DMEM containing 10% FBS and 1% antibiotics–antimycotics. OCR was determined using a seahorse XFe96 or XFp analyzer (Agilent Technologies, Santa Clara, CA, USA) accompanied by an Agilent Seahorse Mito Stress Test kit (Agilent Technologies) according to the manufacturer’s instructions. Key parameters of mitochondrial respiration were analyzed in cells treated with 1 μM oligomycin, 1 μM carbonyl cyanide-4-(trifluoromethoxy) phenylhydrazone (FCCP), and a mixture of 0.5 μM antimycin A/rotenone. At the end of the Seahorse assay, a protein assay was performed to normalize the OCR measurements. OCR values were normalized for the amount of cellular protein in each well.

### 2.9. Caspase Activity Assay

To measure caspase-9 and -3 activity, the cell lysate (20 μg) was mixed with 100 μL of reaction buffer (50 mM HEPES-NaOH [pH 7.4], 10% sucrose, 0.1 percent CHAPS, 10 mM DTT) with 50 μM Ac-LEHD-AFC or 25 μM Ac-DEVD-AMC. The fluorescence generated by cleavage of the fake substrate was monitored every 1 min for 10 min using a Beckman Coulter DTX880 instrument (Beckman Coulter Inc., Fullerton, CA, USA) at excitation and emission wavelengths of 380 and 505 nm for caspase-9 and 360 and 465 nm for caspase-3.

### 2.10. Terminal Deoxynucleotidyl Transferase-Mediated dUTP Nick end Labeling (TUNEL) Assay

Cells were rinsed twice with PBS, and fixed with 4% paraformaldehyde for 15 min at room temperature. After 20 min of permeabilization in 0.25 percent Triton-X100 at room temperature, the TUNEL test kit-FITC (ab66108, Abcam, Cambridge, UK) was used to mark apoptotic cells. The fluorescence signal was obtained using the LSM 880 AiryScan (Carl Zeiss, Göttingen, Germany ). Fluorescence intensity was measured and visualized using NIS-Elements software (Nikon, Tokyo, Japan).

### 2.11. Statistical Analysis

All experiments were repeated at least three times. Comparisons of data between groups were performed by a one-way ANOVA test for multiple-group comparisons. A *p*-value less than 0.05 was considered statistically significant.

## 3. Results

### 3.1. Without PrxIII, Mitochondrial H_2_O_2_ Levels Increase Following Diclofenac Treatment of HepG2 Cells

**Figure 1:**
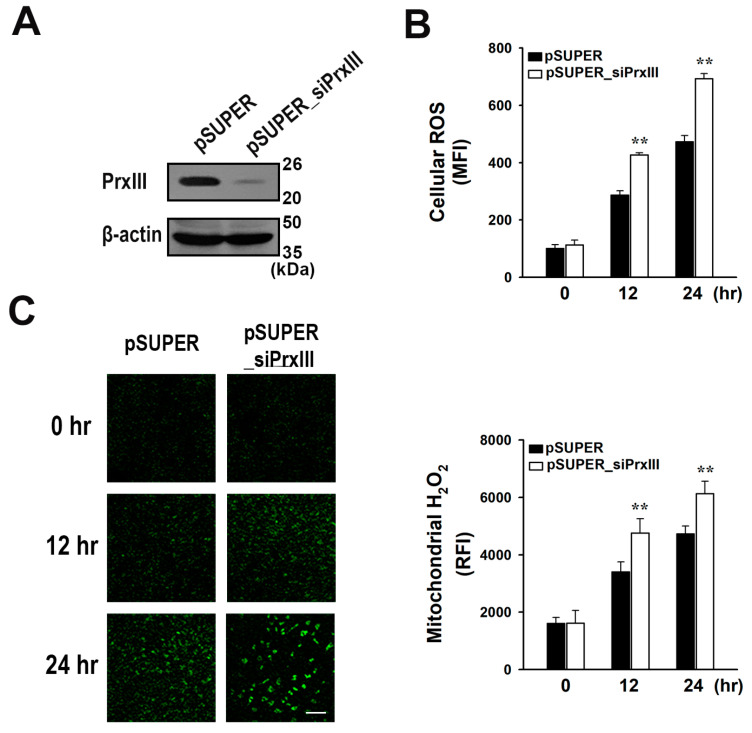
Effects of peroxiredoxin III (PrxIII) knockdown on the accumulation of mitochondrial H_2_O_2_ in human hepatoma cells after exposure to diclofenac. (**A**) Western blotting of lysates from control (*pSUPER*) and PrxIII-knockdown (*pSUPER_siPrxIII)* HepG2 human hepatoma cells. (**B**) Cells were treated with 500 μM diclofenac for the indicated times and cells were stained with CM-H2DCFDA. Cellular ROS levels were measured by quantifying the mean fluorescence intensity (MFI) of CM-DCF using flow cytometry. (**C**) After being treated with 500 μM diclofenac for the indicated times, cells were stained with MitoPY1 which detects mitochondrial H_2_O_2_. Fluorescent images were obtained and quantified at four regions randomly selected on each dish. The relative fluorescence intensity (RFI) was normalized to the number of cells in each image. Scale bar = 100 μm. Mitochondrial H_2_O_2_ levels are shown quantitatively as mean ± standard deviation (n = 5) of RFI. ** *p* < 0.01 versus pSUPER.

Previous research has shown that diclofenac increases the levels of mitochondrial ROS and causes oxidative mitochondrial injury in hepatocytes [2,4]. PrxIII is the primary mitochondrial antioxidant enzyme responsible for H_2_O_2_ degradation in the majority of cell types [12,17]. To investigate the specific importance of mitochondrial H_2_O_2_ during diclofenac-induced mitochondrial oxidative damage in hepatocytes, stable control and PrxIII-depleted cell lines were generated. We transfected HepG2 human hepatoma cells with the pSUPER_siPrxIII vector, which generates small interfering RNAs specific to PrxIII. Cells were transfected with a pSUPER empty vector as a control. Figure 1A depicts PrxIII expression. HepG2 cells stably transfected with the pSUPER and pSUPER_siPrxIII vectors are hereafter referred to as pSUPER and pSUPER_siPrxIII cells, respectively. Figure 1B illustrates that PrxIII depletion substantially increases cellular ROS levels 12 and 24 h after treatment with 500 μM diclofenac. Dichlorodihydrofluorescein CM-H_2_DCFDA, which lacks specificity for individual oxidants [18], was used to determine the overall ROS levels in cells (Figure 1B).

Diclofenac-treated cells were then probed with the fluorescent probe MitoPY1, which responds preferentially to mitochondrial H_2_O_2_ [19]. At 12 and 24 h after diclofenac treatment, the pSUPER_siPrxIII cells exhibited a significantly greater increase in mitochondrial H_2_O_2_ levels in comparison to the pSUPER controls (Figure 1C). These results suggest that in diclofenac-treated hepatocytes, PrxIII depletion results in an accumulation of mitochondrial H_2_O_2_.

### 3.2. PrxIII Depletion Increases Diclofenac-Induced Mitochondrial Oxidative Injury in HepG2 Cells

**Figure 2:**
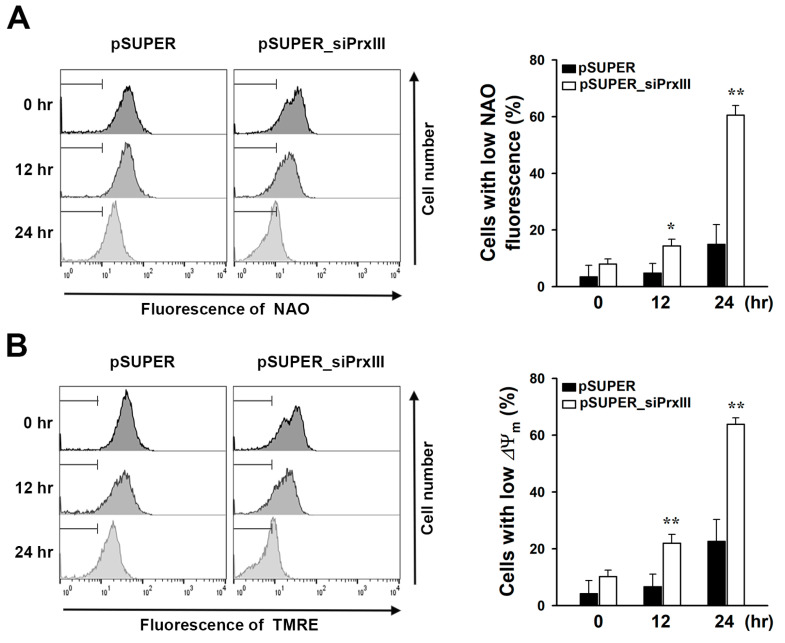
Effects of PrxIII knockdown on mitochondrial membrane potential (ΔΨm) and oxidation of cardiolipin in the mitochondrial membrane of HepG2 hepatoma cells after exposure to diclofenac. Control (*pSUPER*) and PrxIII-knockdown (*pSUPER_siPrxIII)* HepG2 cells exposed to 500 μM diclofenac were incubated for the indicated times. The cells loaded with 10-N-nonyl-acridine orange (NAO) (**A**) or tetramethylrhodamine ethyl ester (TMRE) (**B**) were analyzed by flow cytometry. Representative histograms are shown. Quantitative data are shown as mean ± standard deviation (n = 3) of the percentage of cells with low NAO (**A**) or low ΔΨm fluorescence (**B**). * *p* < 0.05; ** *p* < 0.01 versus pSUPER.

Given that mitochondrial ROS accumulation has been linked to diclofenac-induced mitochondrial oxidative stress and damage [4,20], we investigated oxidative modification of the inner mitochondrial membrane phospholipid cardiolipin. 10-N-nonyl-acridine orange (NAO), which selectively binds to mitochondrial cardiolipin but not other phospholipid forms or oxidized cardiolipin, was used to measure cardiolipin oxidation levels [21,22]. A more pronounced decrease in NAO-stained mitochondria following diclofenac treatment was observed in pSUPER_siPrxIII cells compared with pSUPER cells (Figure 2A). In addition, tetramethylrhodamine ethyl ester (TMRE) was used to measure the change in mitochondrial membrane potential (ΔΨm), which fluoresces in response to ΔΨm-driven mitochondrial uptake [23]. Flow cytometric analysis revealed that diclofenac-induced ΔΨm dissipation was substantially greater in pSUPER_siPrxIII cells than in pSUPER cells (Figure 2B). These results imply that diclofenac-induced increases in mitochondrial H_2_O_2_ play a crucial role in causing mitochondrial oxidative damage.

### 3.3. PrxIII Depletion Exacerbates Mitochondrial Dysfunction Induced by Diclofenac in HepG2 Cells

**Figure 3:**
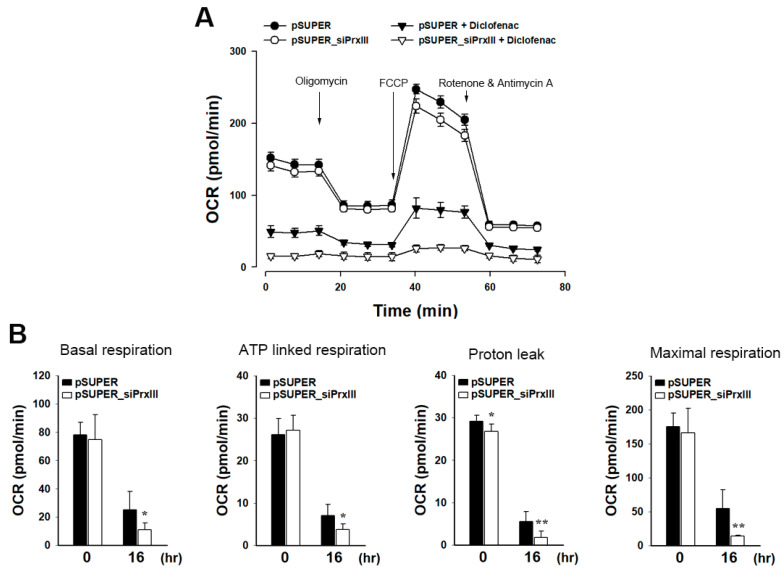
Effects of PrxIII knockdown on respiration of human hepatoma cells after exposure to diclofenac. Control (*pSUPER*) and PrxIII-knockdown (*pSUPER_siPrxIII)* HepG2 hepatoma cells were treated with 500 μM diclofenac for 16 h. (**A**) Representative tracing of the oxygen consumption rate (OCR). Arrows indicate time points when cells were treated with oligomycin, carbonyl cyanide-4-(trifluoromethoxy)phenylhydrazone (FCCP), and rotenone plus antimycin A, respectively. Data are shown as mean ± standard deviation (n = 6). (**B**) The OCR is calculated as basal respiration, ATP linked respiration, proton leak, and maximal respiration. Data are shown as mean ± standard deviation (n = 6). * *p* < 0.05; ** *p* < 0.01 versus pSUPER.

Because PrxIII depletion increases diclofenac-induced oxidative damage to a mitochondrial lipid and ΔΨm dissipation in HepG2 cells (Figure 2), we investigated the potential impact of PrxIII depletion on mitochondrial dysfunction following diclofenac treatment using extracellular flow analysis to quantify mitochondrial activity by observing the oxygen consumption rate (OCR). Bioenergetics analysis with oligomycin, carbonyl cyanide-4-(trifluoromethoxy)phenylhydrazone (FCCP), and rotenone/antimycin A revealed altered cellular metabolic processes and the OCR in HepG2 cells (Figure 3A).

Oligomycin inhibited the activity of ATP synthase and decreased the flux of electrons through the ETC. This resulted in a decline in the OCR, which is linked to ATP production in cells (Figure 3B, ATP-linked respiration). In the presence of oligomycin, the remaining mitochondrial oxygen consumption is proportional to the rate of proton leakage across the inner mitochondrial membrane (Figure 3B, proton leak). The addition of the protonophore FCCP increased the membrane’s proton conductance artificially. As a result, electron transport through the ETC was unhindered and maximal oxygen consumption occurred (Figure 3B, maximum respiration). All mitochondrial-mediated respiration was halted, and only non-mitochondrial respiration persisted after complexes I and III were inhibited with rotenone/antimycin A. Non-mitochondrial respiration was subtracted from baseline respiration to calculate basal respiration (Figure 3B, basal respiration). At 24 h post exposure to diclofenac, mitochondrial respiration significantly decreased as a result of lower basal and maximal respiration, which was accompanied by lower proton leakage that impacted ATP synthesis. Diclofenac significantly reduced mitochondrial respiration in pSUPER_siPrxIII cells compared to pSUPER cells at every phase tested. These results provide further evidence that mitochondrial H_2_O_2_ produced in response to diclofenac causes mitochondrial dysfunction, and that PrxIII protects hepatocytes from diclofenac-induced mitochondrial dysfunction. These results indicate that the elevated levels of mitochondrial H_2_O_2_ caused by PrxIII depletion in diclofenac-treated hepatocytes are closely associated with the induction of mitochondrial dysfunction.

### 3.4. PrxIII Depletion Promotes Apoptosis Induced by Diclofenac in HepG2 Cells

**Figure 4:**
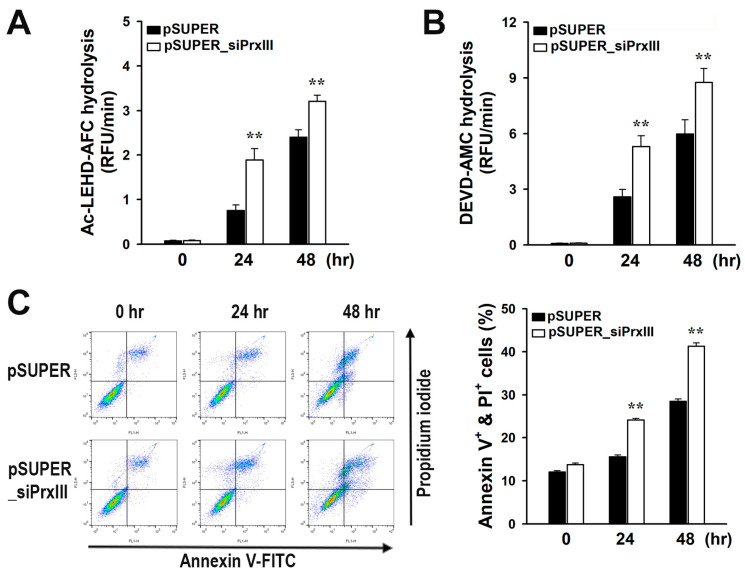
PrxIII knockdown affects mitochondria-mediated apoptosis in human hepatoma cells after exposure to diclofenac. Control (*pSUPER*) and PrxIII-knockdown (*pSUPER_siPrxIII)* HepG2 hepatoma cells were treated with 500 μM diclofenac and incubated for the indicated times. The activity of caspase-9 (**A**) or caspase-3 (**B**) in cell lysates was measured using peptide conjugated to fluorophores. Data are expressed as mean ± standard deviation (n = 5) of the relative fluorescence unit (*RFU*) per min. (**C**) Following analysis by flow cytometry, apoptotic cell death percentage was measured by calculating the sum of annexin V-positive cells, propidium iodide (*PI*)-positive cells, and cells double-positive for annexin V and PI; mean ± standard deviation (n = 5). ** *p* < 0.01 versus pSUPER.

To see if the increased oxidative stress caused by PrxIII deficiency affected the cellular apoptotic pathway, we first evaluated caspase activity with peptides coupled to fluorophores. Caspase-9 and caspase-3 were more activated by diclofenac when PrxIII was depleted (Figure 4A,B). We subsequently investigated the effect of PrxIII depletion on diclofenac-induced apoptotic cell death, which coincided with an increase in caspase activity. Apoptotic cell populations were examined using PI and Annexin V labeling and flow cytometry. Diclofenac administration led to a time-dependent rise in apoptotic cells, with pSUPER_siPrxIII cells exhibiting much more apoptosis than pSUPER cells (Figure 4C). These results suggest that diclofenac-induced increases in mitochondrial H_2_O_2_ play a crucial role in hepatocellular apoptosis, and that PrxIII depletion increases the diclofenac-induced apoptosis in hepatocytes.

### 3.5. Mitochondrion-Targeted Catalase Expression Alleviates the Diclofenac-Induced Apoptosis Amplified in PrxIII-Depleted HepG2 Cells

**Figure 5:**
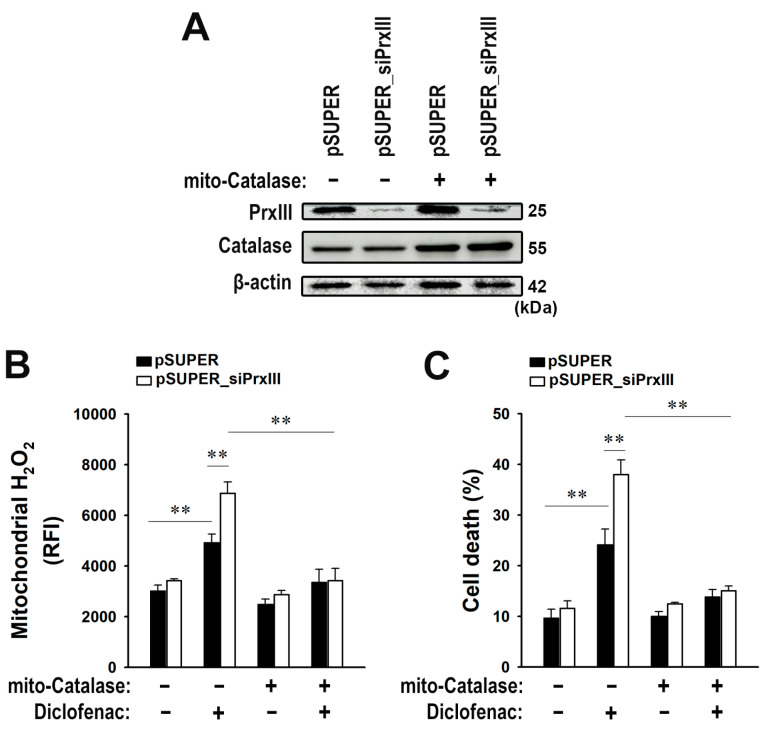
Mitochondrion-targeted catalase expression alleviates the diclofenac-induced apoptosis amplified in PrxIII-depleted HepG2 cells. Control (*pSUPER*) and PrxIII-knockdown (*pSUPER_siPrxIII)* HepG2 hepatoma cells were infected with adenovirus encoding mitochondrion-targeted catalase (*mito-Catalase*) and cultured for 24 h. (**A**) Cell lysates were subjected to immunoblot with the specific antibodies to PrxIII, catalase, and β-actin. The blots are representative of three independent experiments. (**B**) Cells exposed to 500 μM diclofenac for 16 h were stained with MitoPY1. Fluorescent images were obtained and quantified at four regions randomly selected on each dish. Mitochondrial H_2_O_2_ levels are shown quantitatively as mean ± standard deviation (n = 5; ** *p* < 0.01) of the relative fluorescence intensity (RFI). (**C**) Cells were exposed to 500 μM diclofenac for 48 h. Following analysis by flow cytometry, apoptotic cell death percentage was measured by calculating the sum of annexin V-positive cells, propidium iodide (*PI*)-positive cells, and cells double-positive for annexin V and PI; mean ± standard deviation (n = 3; ** *p* < 0.01).

To verify that diclofenac-induced hepatocellular injury was primarily due to accumulation of mitochondrial H_2_O_2_ rather than that of other ROS, we expressed in the pSUPER_siPrxIII HepG2 cells a form of human catalase that is targeted to mitochondria (mito-Catalase) [24]. Catalase’s sole catalytic activity is the conversion of H_2_O_2_ to H_2_O and O_2_. Expression of mito-Catalase was achieved by infection of cells with recombinant adenovirus (Figure 5A). The effects of mito-Catalase on the level of mitochondrial H_2_O_2_ were measured with the use of MitoPY1 and flow cytometry. As demonstrated in Figure 1C, the level of mitochondrial H_2_O_2_ after diclofenac treatment was greater in pSUPER_siPrxIII HepG2 cells than control cells (Figure 5B). Expression of mito-Catalase in the pSUPER_siPrxIII cells, however, reduced the diclofenac-induced accumulation of mitochondrial H_2_O_2_ to a level similar to that apparent in pSUPER controls. The enhanced effects of diclofenac on apoptosis observed in pSUPER_siPrxIII HepG2 cells compared with controls were also significantly reduced after mito-Catalase expression (Figure 5C). These findings indicate that the heightened damage caused by diclofenac in HepG2 cells devoid of PrxIII is due to the accumulation of H_2_O_2_ within the mitochondria, which is caused by the loss of PrxIII activity.

### 3.6. Diclofenac-Induced Apoptosis of Primary PrxIII^−/−^ Murine Hepatocytes Is Significantly Suppressed by Mitochondria-Specific Elimination of H_2_O_2_

**Figure 6:**
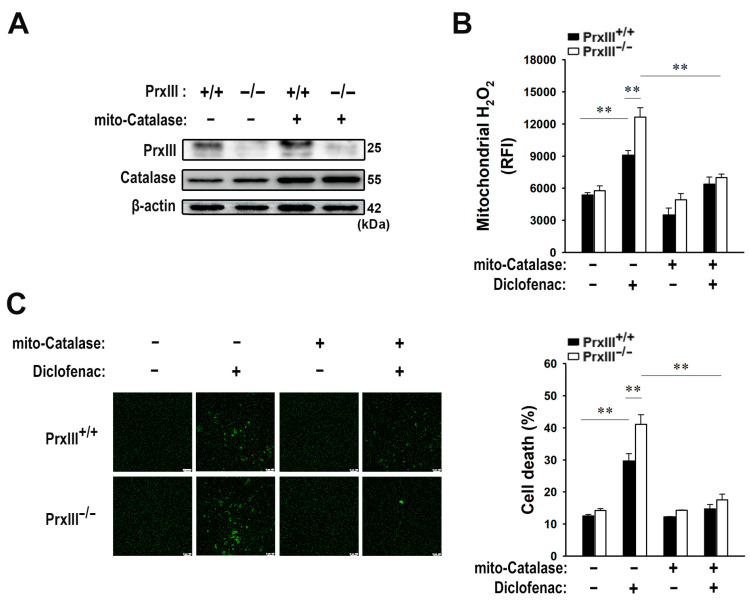
Diclofenac-induced apoptosis of primary PrxIII^−/−^ murine hepatocytes is significantly suppressed by mitochondria-specific elimination of H_2_O_2_. PrxIII^+/+^ and PrxIII^–/–^ mice were infected with adenovirus encoding mitochondrion-targeted catalase (*mito-Catalase*) and cultured for 24 h. (**A**) *Cell* lysates were subjected to immunoblot with the specific antibodies to PrxIII, catalase, and β-actin. The blots are representative of three independent experiments. (**B**) Cells exposed to 500 μM diclofenac for 16 h were stained with MitoPY1. Fluorescent images were obtained and quantified at four regions randomly selected on each dish. Mitochondrial H_2_O_2_ levels are shown quantitatively as mean ± standard deviation (n = 5; ** *p* < 0.01) of the relative fluorescence intensity (RFI). (**C**) Cells were exposed to 500 μM diclofenac for 48 h. The terminal deoxynucleotidyl transferase-mediated dUTP nick end labeling (TUNEL) assay was performed to assess apoptosis. Fluorescent images were obtained and quantified at five regions randomly selected on each dish. Scale bar = 100 μm. Apoptotic cell death was measured as the percentage of TUNEL-positive cells; mean ± standard deviation (n = 3; ** *p* < 0.01).

To demonstrate the crucial role of mitochondrial H_2_O_2_ in diclofenac-induced apoptosis of hepatocytes, primary hepatocytes were isolated from PrxIII^+/+^ and PrxIII^–/–^ mice [13] (Figure 6A). Mitochondrial H_2_O_2_ levels were considerably greater in PrxIII^−/−^ hepatocytes than in PrxIII^+/+^ hepatocytes 16 h after diclofenac treatment (Figure 6B). In accordance with an increase in mitochondrial H_2_O_2_, PrxIII^−/−^ hepatocytes exhibited significantly increased apoptotic cell death 48 h after diclofenac treatment (Figure 6C). We further expressed mito-Catalase in the primary PrxIII^−/−^ mouse hepatocytes (Figure 6A) to confirm that the diclofenac-induced hepatocyte injury was caused primarily by the accumulation of mitochondrial H_2_O_2_ rather than that of other ROS. Mito-Catalase expression in PrxIII^−/−^ hepatocytes reduced diclofenac-induced buildup of mitochondrial H_2_O_2_ to levels comparable to PrxIII^+/+^ hepatocytes (Figure 6B). Mito-Catalase expression additionally lowered diclofenac’s enhanced impact on apoptosis in PrxIII^−/−^ hepatocytes compared to PrxIII^+/+^ (Figure 6C). These findings indicate that among ROS, mitochondrial H_2_O_2_ is a critical component contributing to diclofenac-induced hepatocellular injury, implying that PrxIII can protect hepatocytes against diclofenac-induced apoptosis.

## 4. Discussion

Oxidative stress, a condition marked by an imbalance between ROS production and the cellular antioxidant defense, plays a pivotal role in various pathological processes, particularly in drug-induced hepatotoxicity. Diclofenac is the NSAID most often associated with reports of adverse effects linked to liver injury, specifically hepatocellular and cholestatic forms of liver disease that can ultimately lead to liver failure [25,26]. When given in therapeutic amounts to healthy adults, diclofenac plasma concentration ranged from 2 to 25 μM, depending on dose and administration method [27,28]. However, liver diseases and hepatic cirrhosis impair first-pass metabolism and biotransformation, increasing it [29]. Research indicates that diclofenac overdose, exceeding 1500 mg, increases plasma concentration to 200 μM [30]. Overdose is common with NSAIDs due to their widespread use and over-the-counter availability [31,32]. The acute effect of diclofenac was observed in various culture cells, with IC_50_ values of 392 μM and 331 μM for rat and human primary hepatocytes, respectively, and 763 μM for HepG2 hepatoma cells [33]. In this study, we used diclofenac doses that reflect its acute toxicity.

ROS generation and mitochondrial oxidative damage are the beginning steps in diclofenac-induced liver injury [2,3,4,5,7]. PrxIII is the primary mitochondrial antioxidant enzyme responsible for H_2_O_2_ elimination [12,17]. This study explored the central role of mitochondrial H_2_O_2_ and the increased damage due to loss of PrxIII in the context of diclofenac-induced mitochondrial oxidative damage in hepatocytes by examining the consequences of PrxIII deficiency. Our results show that, among the different cellular ROS produced by diclofenac, mitochondrial H_2_O_2_ is the predominant ROS contributing to hepatocellular toxicity. PrxIII deficiency aggravates mitochondrial oxidative stress and leads to increased hepatocyte apoptosis, implying that PrxIII functions to protect hepatocytes from diclofenac-induced harm by scavenging mitochondrial H_2_O_2_. Our findings suggest that the development of focused therapies aimed at eliminating mitochondrial H_2_O_2_ may reduce the hepatotoxicity of NSAIDs.

The majority of O_2_^•−^ produced by mitochondria is vectorially discharged into the mitochondrial matrix, where intramitochondrial SODs convert it to H_2_O_2_. While not a potent oxidant, O_2_^•−^ does hinder the functionality of mitochondria through the oxidation of the Fe-S centers present in numerous enzymes. Additionally, O_2_^•−^ may combine with nitric oxide generated by mitochondrial nitric oxide synthase [34,35,36] to produce peroxynitrite, an extremely powerful oxidant. The participation of O_2_^•−^ in diclofenac-induced hepatocellular damage and the inhibition of such participation by mitochondrial SODs have been demonstrated [4]. However, while mitochondrial SOD alleviates oxidative stress caused by O_2_^•−^, it also produces H_2_O_2_, which is a distinct form of oxidative stress. H_2_O_2_ is stable enough to diffuse out of mitochondria and have effects outside the mitochondria, despite its significantly higher concentration within mitochondria compared to that in the cytosol. Mitochondrial matrix H_2_O_2_ is reduced by peroxidases, including glutathione peroxidase 1 and 4, and PrxIII and V [12,37]. Glutathione peroxidase 4 specializes in the breakdown of phospholipid hydroperoxides [38]. PrxIII is considerably more prevalent than glutathione peroxidase 1 and PrxV in the majority of tissues and thus is primarily accountable for the scavenging of H_2_O_2_ from mitochondria [12,39]. The balance between the removal and production of H_2_O_2_ in mitochondria must be tightly regulated, but our results suggest that a PrxIII defect tips the scales in favor of production in hepatocytes, and to a greater extent in hepatocytes subjected to triggers of apoptosis such as diclofenac. In addition to H_2_O_2_, PrxIII acts on free or lipid-bound fatty acid hydroperoxides [40]. PrxIII is also an efficient mitochondrial peroxynitrite reductase [41].

Notwithstanding these additional functions, it is probable that the observed consequences of diclofenac-induced hepatocellular injury due to PrxIII deficiency in the current investigation were primarily due to the accumulation of mitochondrial H_2_O_2_, as these effects were eliminated by mito-Catalase overexpression, which converts H_2_O_2_ to H_2_O and O_2_. Catalase is an enzyme that can eliminate H_2_O_2_. However, it is typically found only in the peroxisome of the cell and does not have a substantial impact on the elimination of mitochondrial H_2_O_2_ [42]. However, mito-Catalase [24] is a human catalase with a SOD2 mitochondria leader sequence, thus going to the mitochondria and exhibiting its catalase role. We can demonstrate our hypothesis that mitochondrial H_2_O_2_ is the primary culprit for diclofenac hepatotoxicity. The administration of diclofenac to hepatocytes results in the permeabilization of the mitochondrial membrane through the activation of mitochondria-initiated apoptotic pathways [2,43]. This process is mediated by the opening of the permeability transition pore in the inner membrane and the formation of protein-permeable channels by Bcl-2 family proteins (Bax and Bid) in the outer membrane. The release of proapoptotic proteins and the start of the caspase cascade are associated with an increase in mitochondrial membrane permeability. However, cytochrome c molecules that are tightly bound to the inner mitochondrial membrane by cardiolipin, an anionic phospholipid found mostly in the inner mitochondrial membrane, may not be released by permeabilization of the mitochondrial membranes alone [44,45,46]. Our results indicate that opening of the permeability transition pore, as reflected in a loss of ΔΨm, was enhanced in PrxIII-depleted hepatocytes. Furthermore, this study explores the cascading effects of PrxIII depletion on diclofenac-induced mitochondrial oxidative injury, unveiling a pronounced increase in cardiolipin oxidation levels. Cardiolipin peroxidation facilitates cytochrome c dissociation [47,48], and ^•^OH but not H_2_O_2_ causes lipid peroxidation. Thus, a higher abundance of ^•^OH may be produced by the increased buildup of H_2_O_2_ in PrxIII-depleted hepatocytes. This could then account for the elevated amounts of cardiolipin peroxidation and mitochondria-initiated caspase activation found in diclofenac-treated hepatocytes.

Importantly, functional implications of mitochondrial dysfunction were elucidated through extracellular flux analysis, demonstrating a significant reduction in mitochondrial respiration in PrxIII-depleted cells following diclofenac exposure. This damage is also plausibly caused by oxidation by ^•^OH and could potentially impair the functionality of proteins that are involved in the respiratory chain, such as complexes I and III [7,8].

Diclofenac induces mitochondrial ROS-mediated suppression of mitophagy, which aggravates oxidative stress and thus leads to hepatotoxicity [4]. Despite the increased mitochondrial damage caused by PrxIII deficiency, mitophagy is significantly reduced in the hearts of PrxIII-deficient mice, which exacerbates mitochondrial oxidative damage and ultimately results in heart failure [14]. Therefore, the effective removal of mitochondrial H_2_O_2_ by PrxIII is likely an important protective mechanism that limits dysfunctional mitochondria from creating a vicious cycle comprising mitochondrial damage, mitochondrial ROS production, mitophagy suppression, and accumulation of damaged mitochondria to produce additional mitochondrial ROS that leads to hepatotoxicity. Further studies on the protective role of PrxIII against diclofenac-induced mitophagy impairment will provide insight into understanding the diclofenac-induced liver injury.

While diclofenac causes accumulation of mitochondrial ROS, no previous study has yet provided evidence of a causative relationship between H_2_O_2_ production in the mitochondria and apoptosis. Therefore, it remains unclear if H_2_O_2_ generation is a passive process brought on by the protective effect of mitochondrial SODs or if H_2_O_2_ plays a vital role in the hepatocellular apoptotic process induced by diclofenac. Our findings now support the latter option. This study reveals an exacerbated response to diclofenac-induced apoptosis in PrxIII-deficient hepatocytes, marked by heightened caspase activity and increased apoptotic cell populations. Moreover, to ascertain the pivotal role of mitochondrial H_2_O_2_ in diclofenac-induced hepatocellular apoptosis, mitochondrion-targeted catalase expression was employed. This intervention successfully alleviated the amplified apoptosis observed in PrxIII-depleted HepG2 cells and in primary PrxIII^−/−^ murine hepatocytes, substantiating the notion that diclofenac-induced apoptosis is primarily attributed to the intramitochondrial accumulation of H_2_O_2_ resulting from PrxIII deficiency.

## 5. Conclusions

This study provides strong evidence that, among the different cellular ROS produced by diclofenac, mitochondrial H_2_O_2_ is a key player in diclofenac-induced hepatocellular injury, leading to mitochondrial dysfunction and apoptosis. PrxIII acts as a critical antioxidant enzyme that controls the amount of H_2_O_2_ in mitochondria to lessen the harmful effects of diclofenac. These findings provide important insights into the mechanisms of NSAID-induced liver toxicity and suggest that PrxIII or other antioxidants targeting mitochondrial H_2_O_2_ could be explored as potential therapeutic strategies to protect against hepatotoxicity associated with NSAID use.

## Data Availability

Data are contained within the article.
